# Survival and inactivation kinetics of *Salmonella enterica* serovar Typhimurium in irradiated and natural poultry litter microcosms

**DOI:** 10.1371/journal.pone.0267178

**Published:** 2022-04-19

**Authors:** Alan Gutierrez, Keith R. Schneider

**Affiliations:** 1 Department of Animal Sciences, Emerging Pathogens Institute, University of Florida, Gainesville, FL, United States of America; 2 Food Science and Human Nutrition Department, University of Florida, Gainesville, FL, United States of America; University of Hong Kong, HONG KONG

## Abstract

The use of poultry litter as a biological soil amendment presents a risk for the preharvest contamination of fresh produce by *Salmonella*. In order to properly assess this risk, it is important to understand the factors influencing the persistence of *Salmonella* in poultry litter. This research was performed to investigate the influence of indigenous microflora on the survival of *Salmonella* Typhimurium in poultry litter. Microcosms of irradiated (sterilized) and natural poultry litter were inoculated with *S*. Typhimurium, adjusted to pH 8.0, 0.92 water activity (a_w_), and stored at 30°C for 6 days. *S*. Typhimurium populations (log CFU g^-1^) declined in both litter treatments and there were no significant differences (*P* > 0.05) in recovery between litter treatments on any sampling days (0 to 6). The pH of the natural litter significantly increased (*P* < 0.05) from 8.42 on day 0 to 9.00 on day 6. By day 6, *S*. Typhimurium populations in both litter treatments fell below the limit of detection (1 log CFU g^-1^). The inactivation kinetics of *S*. Typhimurium in both litter treatments were described by the Weibull model. Under the experimental conditions (pH 8.0, 0.92 a_w_, 30°C), the presence or absence of poultry litter microflora did not significantly influence the survival of *S*. Typhimurium. This study demonstrates that the mere presence of poultry litter microflora will not inhibit *Salmonella* survival. Instead, inhibitory interactions between various microorganisms in litter and *Salmonella* are likely dependent on more favorable environmental conditions (e.g., a_w_, pH) for growth and competition.

## Introduction

The poultry industry in the United States (U.S.) produces an estimated 14 million tons of poultry litter and manure each year [1]. Poultry litter is a mixture of poultry excreta, feathers, wasted feed, and bedding materials [2, 3]. Litter is commonly applied to agricultural lands as an organic fertilizer to recycle nutrients such as nitrogen, phosphorus, and potassium [4]. However, it is also a known source of human pathogens such as *Salmonella enterica*, *Listeria monocytogenes*, and *Campylobacter jejuni* [2, 3]. The use of animal manure products, such as poultry litter, is recognized as a major pathway for the preharvest contamination of fresh produce [5]. Several studies have demonstrated *Salmonella*’s survival in field soils amended with poultry manure or litter, and the potential for contamination of fresh produce grown in amended soils [6–9].

To accurately assess the risks of using poultry litter as a biological soil amendment, it is important to understand how long *Salmonella* may survive in litter before it is applied to fields. The survival of *Salmonella* in poultry litter depends on various extrinsic (e.g., litter treatments, temperature) and intrinsic factors (e.g., pH, microflora) [10–13]. Laboratory studies have observed *Salmonella*’s survival in poultry litter varying from 2 days [14] to 18 months [15] depending on these factors. Further studies of poultry litter have identified pH, moisture content, water activity (a_w_), ammonia, and microflora as major intrinsic factors influencing the survival of *Salmonella* [12–14, 16, 17].

The microflora of poultry litter has been well characterized in numerous studies [18–25]. Poultry litter may contain bacterial populations as high as 10^11^ CFU g^-1^ [19, 20, 26]. However, the influence of this microflora on *Salmonella*’s survival in litter is not well understood. Studies on the practice of re-using poultry litter for multiple flocks have suggested that the reduced prevalence of *Salmonella* in re-used litter is due to competitive exclusion or bacterial antagonism of the microflora in this litter [17, 27, 28]. Few studies have directly investigated the influence of poultry litter microflora on the survival of *Salmonella* [13, 14, 29, 30]. Alexander et al. [29] reported that *Salmonella* survived longer in autoclaved (63 days) than non-autoclaved (29 days) litter samples. Similarly, Erickson et al. [30] observed significantly lower populations of *S*. Enteritidis after three days in non-autoclaved chicken manure compared with autoclaved chicken manure. However, the authors of this study attribute the decline of *Salmonella* populations to an increase in the pH of the non-autoclaved litter to alkaline levels [30]. While autoclaving will reduce microbial populations in poultry litter and manure, it is typically not sufficient to achieve sterilization [30]. Turnbull and Snoeyenbos [14] conducted a series of studies to determine the effects of ammonia, pH, a_w_, and litter microflora on the survival of *Salmonella* in poultry litter. They reported that unfavorable a_w_ levels and high pH, resulting from dissolved ammonia, were the main factors causing *Salmonella* die-off in the litter [14].

Predictive microbiology is an ever-evolving discipline within microbiology that involves the use of mathematical and statistical models to describe and predict microbial behavior [31]. Early predictive models were developed to describe bacterial death kinetics during thermal processing in the food industry. Current modeling methodologies can be used to predict the growth and inactivation of microorganisms under various conditions [32]. Modeling studies of animal manures have typically been applied to understand the transport and fate of pathogens and indicator microorganisms in the environment [33–36]. Studies modeling the survival of *Salmonella* in poultry litter are limited [12, 37, 38]. Whereas several studies focus on modeling the thermal inactivation of *Salmonella* in poultry litter [37, 38], Payne et al. [12] used the Churchill model [39] to describe the growth and inactivation of *Salmonella* in poultry litter under various pH (4, 7, 9) and a_w_ (0.84, 0.91, 0.96) conditions. Further applying modeling methodologies to survival studies in animal manures, such as poultry litter, will help strengthen our understanding of pathogen persistence in these biological soil amendments.

Current research suggests that poultry litter microflora may influence the survival of *Salmonella*, however few studies have directly assessed this potential influence [13, 14, 29, 30]. In this study, poultry litter samples were sterilized via irradiation treatment. Irradiated and natural litter samples were inoculated with *S*. Typhimurium, adjusted to pH 8.0 and a a_w_ of 0.92 based on previous survey studies [40, 41], and stored at 30°C. *Salmonella* populations, pH, and a_w_ were monitored daily for 6 days. Total ammonia nitrogen (TAN) was measured on days 0, 3, and 6. The objective of this study was to assess the influence of the litter microflora’s presence or absence on the survival of *S*. Typhimurium in irradiated and natural poultry litter microcosms. Furthermore, *Salmonella* survival data was fitted using non-linear survival models to describe the inactivation kinetics of both litter treatments.

## Methods and materials

### Poultry litter

Poultry litter was collected from a commercial poultry producer in North Florida. The litter collected had been removed from broiler houses and piled in a covered, two-wall, open air shed. Pine shavings were the bedding material used by this producer. The collected litter was passed through a brass sieve (0.25 in. [6.3 mm]; Fisher Scientific, Fair Lawn, NJ) to remove large clumps and feathers. The microflora of the litter was enumerated, in triplicate, by transferring 10 g of litter into sterile filtered Whirl-Pak^®^ bags (7.5 x 12 in. [19.0 x 30.4 cm]; Nasco, Fort Atkinson, WI) with 90 mL of buffered peptone water (BPW; BD, Difco, Sparks, MD) followed by 1 min homogenization in a Smasher Lab Blender (AES Chemunex, Bruz, France). Ten-fold dilutions were performed in 0.1% peptone water (PW; BD, Difco), and 0.1 mL was plated onto tryptic soy agar (TSA; BD, Difco) and dichloran rose-bengal chloramphenicol agar (DRBC; BD, Difco) to determine aerobic plate counts (APC) and yeast and mold counts (YMC), respectively. The limit of detection (LOD) for APC and YMC was lowered to 1 log CFU g^-1^ by spread plating four 250 μL subsamples from the Whirl-Pak^®^ bags onto TSA and DRBC. The TSA and DRBC plates were incubated at 35°C for 18–24 h and 25°C for 5 days, respectively. To determine the presence or absence of *Salmonella*, the Whirl-Pak^®^ bags were incubated at 37°C for 18–24 h and transferred to selective media. Selective enrichment was performed by transferring 0.1 mL and 1 mL from each bag to 10 mL of Rappaport-Vassiliadis R10 broth (RV; BD, Difco) and tetrathionate broth (TT, Remel, Lenexa, KS), respectively. Both broth enrichments were incubated at 42°C for 24 h in a shaking incubator at 100 rpm. Following selective enrichment, 10 μL was streaked onto xylose lysine deoxycholate agar (XLD; BD, Difco) and incubated at 37°C for 18–24 h. Presumptive *Salmonella* colonies were transferred to TSA and incubated at 37°C for 18–24 h. Presumptive *Salmonella* isolates on TSA were confirmed via agglutination assay with *Salmonella* O Antiserum Poly A-I & Vi (BD, Difco) [42]. A portion of the litter was sent to the Sterigenics irradiation facility (Mulberry, FL) to be sterilized. The litter was irradiated with a minimum dose of 26.04 kGy. The efficacy of the irradiation treatment was confirmed by repeating the microflora enumeration procedures previously described. Both irradiated and natural litter were stored at -20°C during the study.

### Inoculum preparation

A *Salmonella* Typhimurium (ST) isolate previously recovered from poultry litter [41] was used in this study. Stepwise exposures were used to induce antimicrobial resistance in the ST isolate to 200 μg mL^-1^ rifampicin (RIF; Sigma-Aldrich, St. Louis, MO). The ST inoculum was prepared by transferring a frozen culture to 10 mL of tryptic soy broth (TSB; BD, Difco) with 80 μg mL^-1^ RIF and incubating at 37°C for 18–24 h in a shaking incubator at 100 rpm. This overnight culture was transferred once more into 10 mL TSB with 80 μg mL^-1^ RIF and incubated under the same conditions. The final culture was prepared by transferring the overnight culture to 25 mL of TSB with 80 μg mL^-1^ RIF in a 50 mL conical centrifuge tube (Fisher Scientific) and incubating under the same conditions. The final inoculum was prepared by centrifuging (1,789 x g, 10 min) and washing the cells twice with 0.1% PW. The cells were resuspended in 25 mL of 0.1% PW. Enumeration of the inoculum was performed by spreading 0.1 mL of 0.1% PW serial dilutions onto TSA with 80 μg mL^-1^ RIF, in duplicate, and incubating at 37°C for 18–24 h before counting.

### Litter inoculation and adjustment (pH and a_w_)

Two hundred grams of irradiated or natural poultry litter were placed in a sterile sample bag (7 x 12 in. [17.7 x 30.4 cm]; Fisher Scientific) and adjusted to pH 8.0 by adding 2 M HCl or 1 M NaOH. The litter was thoroughly mixed by hand for 2 min after each addition. The litter pH was determined by adding 3 g of litter to 15 mL of deionized water, vortexing for 30 s, and letting stand for 1 min before measuring with a pH probe (model: HI72911B; Hanna Instruments, Smithfield, RI). After adjusting the pH, the litter was inoculated with two consecutive 10 mL aliquots of the ST inoculum. After each inoculum addition, the litter was thoroughly mixed by hand for 2 min. Sterile deionized water was added to adjust the litter a_w_ to 0.92. Litter a_w_ was measured according to the manufacturer’s instructions using an AquaLab Model 4 Water Activity Meter (Decagon Devices Inc., Pullman, WA). The pH of irradiated litter samples was readjusted to 8.0 after being inoculated. Twenty grams of inoculated litter were transferred to six sterile sample bags (4.5 x 9 in. [11.4 x 22.8 cm]; Fisher Scientific) and stored at 30°C.

### Sampling procedure

Litter samples were stored at 30°C and collected on days 0, 1, 2, 3, 4, 5, and 6. Each sample bag was thoroughly mixed by hand for 1 min before sampling. Day 0 litter samples were collected from the inoculation sample bag. To enumerate ST populations, 10 g of litter was added to 90 mL of BPW in a sterile filtered Whirl-Pak bag (7.5 x 12 in. [19.0 x 30.4 cm]) and homogenized for 1 min in a Smasher Lab Blender (AES Chemunex, Bruz, France). Ten-fold dilutions were performed in 0.1% PW, and 0.1 mL was plated onto XLD supplemented with 80 μg mL^-1^ RIF and incubated at 37°C for 18–24 h. When litter samples approached the LOD (1 log CFU g^-1^), four 250 μL subsamples from the Whirl-Pak^®^ bag were plated onto XLD supplemented with 80 μg mL^-1^ RIF. Litter enrichments were performed by incubating the Whirl-Pak^®^ bags for 18–24 h at 37°C, followed by selective enrichment in RV and TT broth and streaking onto XLD as previously described. Presumptive ST isolates were subcultured on TSA, incubated at 37°C for 18–24 h, and confirmed via agglutination assay with *Salmonella* O Antiserum Group B (BD, Difco). Litter pH and a_w_ were determined on each sampling day as previously described. Litter total ammonia nitrogen (TAN) was also sampled on days 0, 3, and 6. Litter samples were stored at -20°C until TAN was measured. To measure litter TAN, 1 g of litter was added to 100 mL of deionized water in a 125 mL Erlenmeyer flask and mixed for 1 h at 175 rpm [43]. The litter solution TAN was measured according to the manufacturer’s instructions for the ammonia combination ion-selective electrode (ISE) (model: HI4101; Hanna Instruments) using a pH/ORP/ISE meter (model: HI98191; Hanna Instruments). Litter TAN was calculated using the following equation (Eq 1), where the final weight (g) is the sum of the deionized water and litter sample weight added into the flask.


$$Litter\;TAN\;(ppm)=soln.TAN\;(ppm)*\frac{final\;weight\;(g)-litter\;sample\;(g)}{litter\;sample\;(g)}$$ (1)

### Statistical analysis

Bacterial plate counts (CFU g^-1^) were log-transformed (log_10_ CFU g^-1^) for statistical analysis. Enrichments were performed when ST populations in litter samples fell below the plating method LOD (10 CFU g^-1^; 1 log CFU g^-1^). Positive enrichments were assigned a value of 5 CFU g^-1^ (0.70 log CFU g^-1^), halfway between zero and the LOD. A two-way ANOVA followed by Tukey’s honest significance test was used to compare mean ST populations, pH, a_w_, and TAN between the litter treatments on each sampling day. Experiments in the irradiated and natural litter were performed in triplicate. Statistical analyses and visualizations were performed in R version 4.0.4 [44] with significance set at α = 0.05.

### Inactivation models, parameter estimation, and goodness-of-fit

Several primary inactivation models were fitted to the survival data in order to determine the best fitting model. The following models were considered: log-linear [45], bilinear (with and without tailing or shoulder effects) [46], Geeraerd (with and without tailing or shoulder effects) [47], Weibull [48], Weibull with tailing effects [49], and double Weibull [50]. ST concentration (log CFU g^-1^) data from each litter treatment were fitted to these models using the nls() function in R [44]. Bootstrapped confidence intervals were generated for the fitted model parameters using the nlsBoot() function from the nlsMicrobio R package [46]. The Akaike information criterion (AIC; Eq 2) [51] and Bayesian information criterion (BIC; Eq 3) [52] were used to assess the goodness-of-fit for the fitted models.


$$AIC=p∙Ln\;\left(\frac{RSS}{p}\right)+2k$$ (2)

$$BIC=p∙Ln\;\left(\frac{RSS}{p}\right)+k∙Ln(p)$$ (3)

In both Eqs (2 and 3), *RSS* is the residual sum of squares, *p* is the number of data points used to fit the model, and *k* is the number of parameters in the model. Lower AIC and BIC scores indicate a better fitting model.

Inactivation kinetics in this study were described by the Weibull model. The Weibull model (Eq 4) is an empirical inactivation model which accounts for the non-linearity of microbial survival curves as an alternative to the classical Bigelow model of first-order kinetics [53].


$$\log_{10}\left(N\right)=\log_{10}(N_{0})-{\left(\frac{t}{\delta }\right)}^{p}$$ (4)

In this model (Eq 4), *N* is the number of survivors (CFU g^-1^), *N*_0_ is the initial inoculum concentration (CFU g^-1^), *t* is the time (days), *p* is the shape of the inactivation curve (dimensionless), and *δ* is the time (days) to the first decimal reduction of the microbial population [53].

## Results

### Litter microflora and physicochemical parameters (a_w_, pH, and TAN)

The natural poultry litter microflora populations were 6.57 ± 0.10 and 3.01 ± 0.03 log CFU g^-1^ for APC and YMC, respectively. After irradiation, no growth was observed (< 1 log CFU g^-1^) on plates for both APC and YMC. No indigenous *Salmonella* were recovered from the natural or irradiated litter.

The a_w_ levels decreased significantly (*P* < 0.05) over the 6-day sampling period by 0.012 and 0.009 for the irradiated and natural litter microcosms, respectively (Table 1). In the irradiated litter, the pH decreased from 7.96 (day 0) to 7.87 (day 6), but this change was not statistically significant (*P* > 0.05). In contrast, there was a significant increase (*P* < 0.05) in the pH of the natural litter from 8.42 (day 0) to 9.00 (day 6). The pH of the natural litter was also significantly higher (*P* < 0.05) than the irradiated litter on all sampling days. The TAN level in both litter treatments did not significantly change during the sampling period (*P* > 0.05) (Table 1).

**Table 1:** Water activity (a_w_), pH, and total ammonia nitrogen (TAN) measures in poultry litter.

		Day						
Measurement	Litter treatment	0	1	2	3	4	5	6
a_w_	Irradiated	0.919 ± 0.002^abc^	0.912 ± 0.002^cdef^	0.912 ± 0.002^cdef^	0.911 ± 0.001^def^	0.911 ± 0.002^def^	0.909 ± 0.001^ef^	0.907 ± 0.002^f^
	Natural	0.925 ± 0.002^a^	0.921 ± 0.002^ab^	0.920 ± 0.002^abc^	0.917 ± 0.001^bcd^	0.918 ± 0.004^abc^	0.915 ± 0.005^bcde^	0.916 ± 0.003^bcde^
pH	Irradiated	7.96 ± 0.05^a^	7.88 ± 0.04^a^	7.86 ± 0.04^a^	7.85 ± 0.03^a^	7.88 ± 0.02^a^	7.86 ± 0.06^a^	7.87 ± 0.04^a^
	Natural	8.42 ± 0.08^b^	8.49 ± 0.03^b^	8.54 ± 0.03^b^	8.76 ± 0.00^c^	8.89 ± 0.05^d^	8.93 ± 0.06^d^	9.00 ± 0.03^d^
TAN (ppm)	Irradiated	1,383 ± 300^abc^	ND	ND	1,640 ± 92^ab^	ND	ND	1,708 ± 49^a^
	Natural	1,255 ± 162^bc^	ND	ND	1,230 ± 56^c^	ND	ND	1,421 ± 52^abc^

Reported values are mean ± standard deviation (n = 3). For each measurement, means with the same letter across rows and columns are not significantly different (*P* > 0.05). ND, not determined.

### *Salmonella* Typhimurium (ST) survival in irradiated and natural poultry litter microcosms

**Fig 1:**
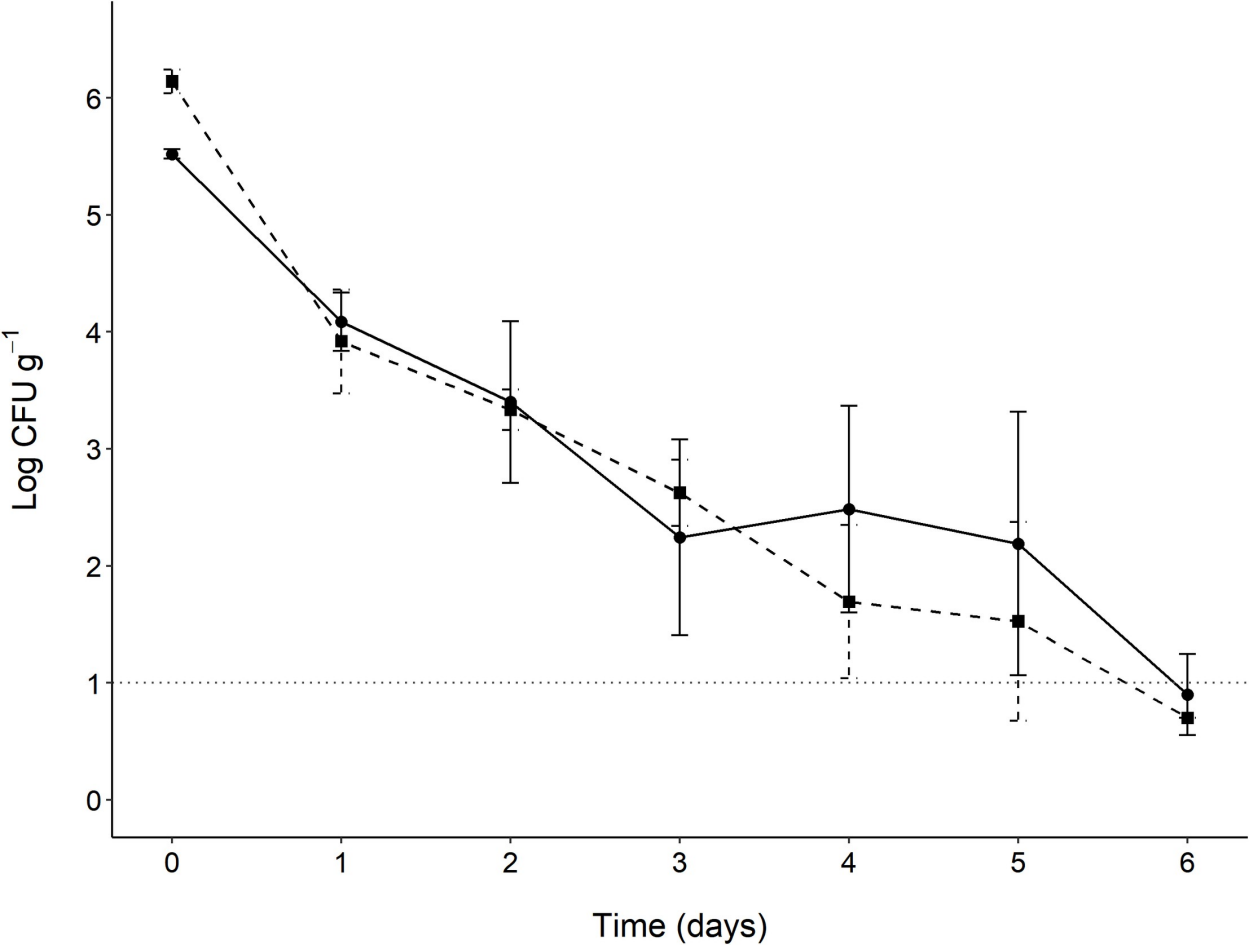
Survival of *Salmonella* Typhimurium (ST) in irradiated (■; dashed line) and natural (●; solid line) poultry litter (pH 8.0, 0.92 a_w_, 30°C). Limit of detection (1 log CFU g^-1^) represented by the dotted line. Data points represent means and error bars represent standard deviations (n = 3).

On day 0, the ST inoculum (log CFU g^-1^) was higher in the irradiated litter (6.14) than the natural litter (5.52), though this difference was not significant (*P* > 0.05) (Fig 1). In the natural litter the ST population increases slightly from day 3 to 4 (2.24 to 2.48 log CFU g^-1^), but this increase was not statistically significant (*P* > 0.05). Between days 4 and 5, ST populations were only reduced by 0.17 and 0.29 log CFU g^-1^ in irradiated and natural litter samples, respectively. On each sampling day, there were no significant differences (*P* > 0.05) between ST populations recovered from both litter treatments (S1 Table). *Salmonella* populations in both litter treatments fell below the LOD (1 log CFU g^-1^) on day 6 (Fig 1).

**Fig 2:**
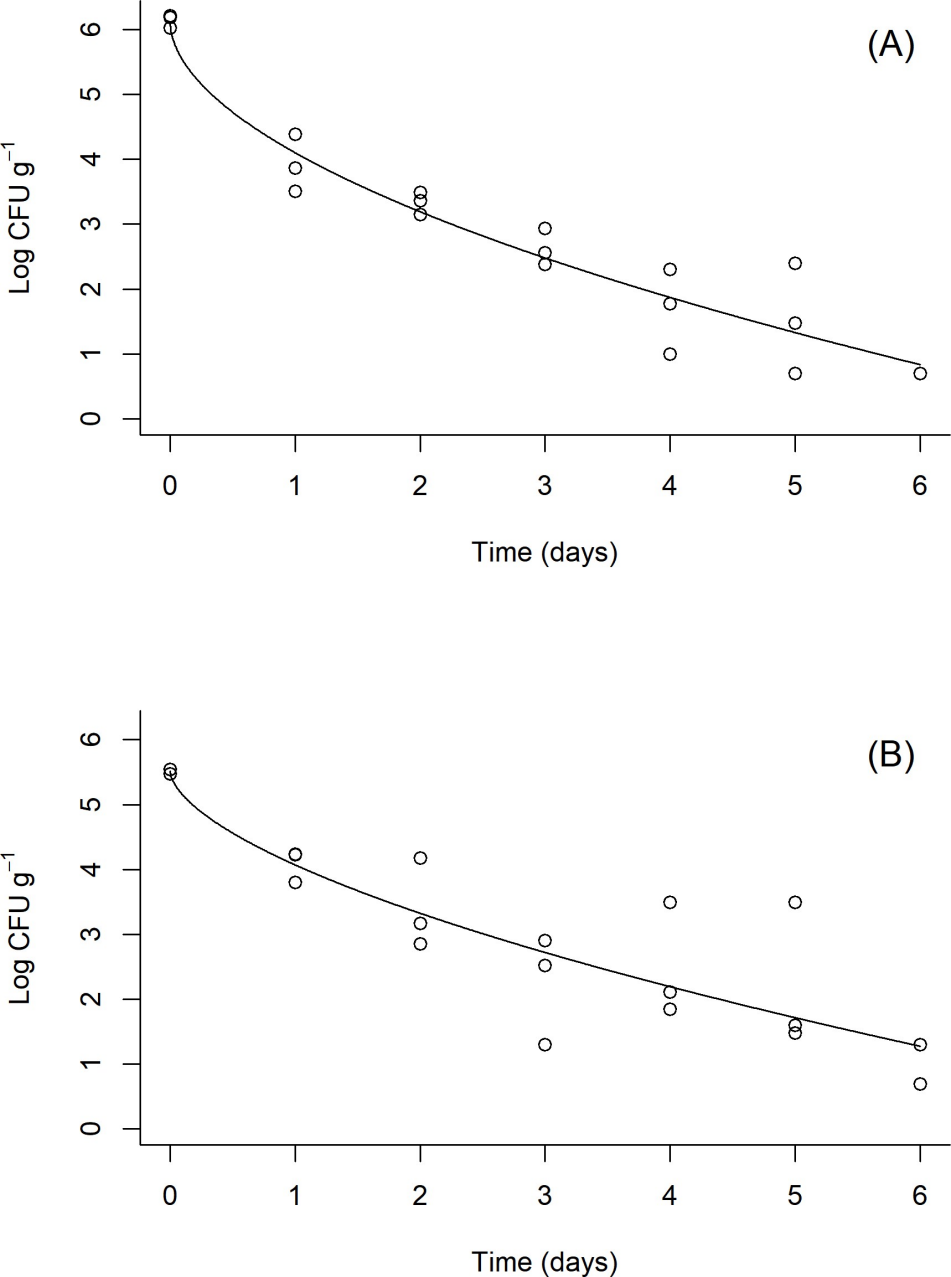
*Salmonella* Typhimurium (ST) survival fitted by Weibull models for irradiated (A) and natural (B) litter microcosm data. Datapoints (°) for each sampling time (n = 3) and Weibull model curve are shown.

Table 2 shows the goodness-of-fit measures (AIC and BIC) for the different inactivation models tested. For both litter treatments, the Geeraerd model without tailing resulted in the lowest AIC and BIC scores. Despite these lower scores, this model is disqualified because its shoulder parameter (Sl), the duration of the shoulder effect, was negative, which is not physically possible [47, 54]. As a result, the inactivation kinetics of ST in both litter treatments was best fitted to the Weibull model. Parameter values for the Weibull model survival curves are presented in Table 3. The fitted survival models for both irradiated and natural litters represent convex curves (*p* < 1) with no shoulder (delayed response) or tailing (stabilized decline) effects (Fig 2). While the *δ* value was lower in the irradiated litter (0.27) than the natural litter (0.58), these parameters were not significantly different according to their 95% confidence intervals (Table 3).

**Table 2:** Goodness-of-fit scores for inactivation models fitted to *Salmonella* Typhimurium (ST) survival data in irradiated and natural litter.

Model	Irradiated litter		Natural litter	
	AIC	BIC	AIC	BIC
Geeraerd without tailing	-32.89	-29.75	-12.14	-9.00
Weibull	-32.06	-28.92	-12.04	-8.91
Geeraerd (shoulder and tailing)	-30.90	-26.72	ND	ND
Geeraerd without shoulder	-20.03	-16.89	-8.24	-5.10
Log-linear	- 17.88	-15.79	-10.21	-8.12
Bilinear without shoulder	-17.25	-14.12	ND	ND
Weibull with tailing	ND	ND	ND	ND
Double Weibull	ND	ND	ND	ND
Bilinear without tailing	ND	ND	ND	ND

ND, not determined (model could not be fitted to the data); AIC, Akaike information criterion; BIC, Bayesian information criterion.

**Table 3:** Parameters values of the fitted Weibull models describing the survival of *Salmonella* Typhimurium (ST) in irradiated and natural poultry litter.

Litter treatment	*N*_0_	*δ*	*p*
Irradiated	6.12 [5.69, 6.57]	0.27 [0.11, 0.53]	0.54 [0.43, 0.68]
Natural	5.45 [4.80, 6.14]	0.58 [0.19, 1.46]	0.61 [0.41, 0.98]

Best-fit parameter values and their 95% confidence intervals, [CI], are reported. *N*_0_, initial inoculum concentration (log CFU g^-1^); *δ*, time (days) to first decimal reduction; *p*, shape of inactivation curve.

## Discussion

Numerous studies of *Salmonella* in poultry litter and manure have suggested that microflora may decrease the prevalence and survival of *Salmonella* via competitive exclusion or bacterial antagonism [20, 27, 28, 55]. Roll et al. [27] and Muniz et al. [28] attributed the decreased prevalence of *Salmonella* in reused poultry litter to the complex microbial communities in reused litter. In their study of litter microflora, Lu et al. [20] identified several bacterial species which may be involved in composting organic matter and suggested that this may explain the absence of human pathogens in certain poultry litters. In laboratory studies, the presence of different genera have been positively and negatively correlated to *Salmonella* populations in the litter [13]. This study sought to determine the influence of litter microflora on *Salmonella* by inoculating ST into poultry litter with (natural) and without (irradiated) microflora present.

Survey studies have reported that *Salmonella* prevalence is highest in poultry litter at a_w_ levels of 0.90–0.95 [16, 56, 57]. Poultry litter studies have frequently observed the average pH of litter to be 8 [40, 41, 56]. Himathongkham et al. [58] inoculated poultry manure with *Salmonella* and reported that a_w_ levels of 0.75–0.89 resulted in a 2 to 3 log CFU g^-1^ reduction within 8 h, and a 6 log CFU g^-1^ reduction within 8 days at 0.89 a_w_. Payne et al. [12] investigated the interaction between a_w_ and pH in poultry litter and reported that *Salmonella* populations were able to grow at pH 7 and 9 with a a_w_ of 0.96, whereas the greatest reductions (5 log CFU g^-1^) occurred at pH 4 with a a_w_ of 0.84. In their study, pH was the dominant factor influencing the survival of *Salmonella*, where *Salmonella* reductions were fastest in pH 4 trials regardless of the a_w_ level (0.84, 0.91, 0.96) [12]. In this study, poultry litter was adjusted to pH 8.0 and a a_w_ of 0.92. Over the 6-day sampling period, there were no significant differences (*P* > 0.05) between the ST populations recovered from the irradiated and natural poultry litter (Fig 1). The presence or absence of the litter microflora did not significantly influence the survival of *Salmonella* as both populations fell below 1 log CFU g^-1^ on day 6. In contrast, Payne et al. [12] recovered *Salmonella* populations at ≥ 1 log CFU g^-1^ for over 40 days, despite conducting their studies under similar conditions (autoclaved litter, pH 7–9, a_w_ 0.91, 30°C). Their study used a cocktail of *Salmonella* serovars (Heidelberg, Newport, Typhimurium) to inoculate their litter, suggesting that differences in serovar survival may be responsible for the greater *Salmonella* persistence they observed [12]. The results of this study suggest that a_w_ was the primary factor contributing to the inactivation of *S*. Typhimurium in the poultry litter microcosms.

Previous studies have suggested that ammonia in poultry litter may contribute to the inactivation of *Salmonella* [14, 27, 59–61]. Ammonia production in litter is greatest at alkaline pH levels, which leads to ammonia volatilization into the atmosphere as NH_3_ gas [62]. Turnbull and Snoeyenbos [14] and Himathongkham et al. [59] showed that NH_3_ gas could be used to reduce *Salmonella* populations in poultry litter and manure. In the present study, the average total ammonia nitrogen (TAN) in both litter samples increased over time, however this increase was not statistically significant (*P* > 0.05) (Table 1). The pH of the poultry litter was adjusted to 8.0 in both litter treatments. The pH of the natural litter increased significantly (*P* < 0.05) to 9.0 on day 6, whereas it did not significantly change in the irradiated litter. In their study, Turnbull and Snoeyenbos [14] attributed the rise of litter pH they observed to increased ammonia dissolved in the litter system. While the increased pH of the natural litter in this study is evidence of microbial activity, it does not appear to be a result of increased TAN. Koziel et al. [63] determined the minimum inhibitory concentration (MIC) of NH_3_ against *S*. Typhimurium to be 0.1 M NH_3_ (2,775 ppm TAN) in phosphate-buffered saline solutions at pH 9.0. In their study, the antimicrobial effects of NH_3_ were greatly reduced at pH levels < 9.0 [63]. With the pH of the litter remaining < 9.0 for most sampling times and the TAN level not increasing significantly, it is unlikely that ammonia had a major influence on the survival of the *Salmonella*.

In this study, modeling was used to provide a quantitative description of *Salmonella*’s inactivation kinetics in the poultry litter microcosms. The Weibull model parameters can be interpreted to provide insights about *Salmonella*’s response to litter conditions. The survival curves for both litter treatments do not exhibit shoulder or tailing effects (Fig 2). A shoulder effect in survival models suggests that the microorganism has some initial resistance to the treatment conditions, which results in a delayed response. A tailing effect indicates a subpopulation that is more resistant to the treatment than the main population, which results in a slowing inactivation rate over time. The absence of a shoulder suggests that the ST inoculum had no resistance to the litter conditions, resulting in an immediate reduction of the population [64–66]. While there are no tailing effects in the presented models, the greater persistence of *Salmonella* observed by Payne et al. [12] suggests that tailing effects are possible in poultry litter under similar conditions. The *p* parameter, which determines whether the Weibull survival curve is concave (*p* > 1) or convex (*p* <1), can be linked to physiological effects caused by microbial stress responses [53, 64]. The *p* parameters of both litter treatments are < 1, which indicates that the remaining *Salmonella* populations at each time point have less probability of dying. This suggests the existence of a more resistant subpopulation of *Salmonella* or that the population is adapting to the stress over time [53]. These stress adaptation responses of *Salmonella* have similarly been reported in studies of *Salmonella*’s persistence in manure-amended soils, which often observe persistent populations in soils for >100 days [9, 67, 68]. Altogether, none of the parameter estimates for both litter models were significantly different. This suggests that the inactivation kinetics of *Salmonella* in either litter was not influenced by the presence or absence of the natural microflora.

This study determined that the presence of poultry litter microflora is not inherently inhibitory to *Salmonella*. With no significant differences in the survival of *Salmonella* in the irradiated and natural litter, the a_w_ level is primarily responsible for the inactivation of *Salmonella* observed in this study. It should be noted that these conclusions and the applicability of the survival models developed are limited to the conditions (pH, a_w_, temperature) specifically set in this study. Survival times may differ if a_w_ levels are sufficient to allow for the growth of *Salmonella* and other microorganisms in the litter. This change would also likely result in more apparent differences between the irradiated and natural litter. Any influence on the survival of *Salmonella* likely depends on complex interactions between different microbial species present in the litter, where the nature of their interactions may be protective or detrimental [13]. Further research concerning the interactions between litter microflora and pathogens, like *Salmonella*, is needed. Widening the use of predictive microbiology in these studies to describe bacterial growth and inactivation kinetics will also allow for deeper understanding of pathogen behavior under various environmental conditions, which will guide the development of improved animal manure treatments and management strategies.

## Supporting information

S1 Table *Salmonella* Typhimurium (ST) populations (log CFU g^-1^) in irradiated and natural poultry litter microcosms. (DOCX)

S2 Table Irradiated litter study dataset. (XLSX)
