# Supplementary material for: Survival and inactivation kinetics of Salmonella enterica serovar Typhimurium in irradiated and natural poultry litter microcosms
Source: PLoS One. 2022 Apr 19;17(4):e0267178. doi: 10.1371/journal.pone.0267178 (PMC9017879; doi:10.1371/journal.pone.0267178)
Supplement: S1 Table — (DOCX) [file pone.0267178.s001.docx]

**SUPPORTING INFORMATION**

**Table S1.** *Salmonella* Typhimurium (ST) populations (log CFU g^-1^) in irradiated and natural poultry litter microcosms

|  | Day | | | | | | | |
| --- | --- | --- | --- | --- | --- | --- | --- | --- |
| Litter treatment | 0 | 1 | 2 | 3 | 4 | 5 | 6 |  |
| Irradiated | 6.14 ± 0.10^a^ | 3.92 ± 0.44^bcd^ | 3.33 ± 0.17^cde^ | 2.62 ± 0.28^cdef^ | 1.69 ± 0.65^efg^ | 1.52 ± 0.85^fg^ | 0.70 ± 0.00^g^ |  |
| Natural | 5.52 ± 0.04^ab^ | 4.09 ± 0.25^bc^ | 3.40 ± 0.69^cde^ | 2.24 ± 0.84^defg^ | 2.48 ± 0.88^cdef^ | 2.19 ± 1.13^defg^ | 0.90 ± 0.35^fg^ |  |

Reported values are mean ± standard deviation of bacterial counts (n = 3). Means with the same letter across rows and columns are not significantly different (*P* > 0.05).
